# Effect of midwife-led continuity of care combined with individualized breast management on postpartum recovery and lactation function in women undergoing cesarean section

**DOI:** 10.3389/fmed.2025.1608027

**Published:** 2025-10-31

**Authors:** Hua Cai, Yan Lu, Xinyi Kang, Liping Chen

**Affiliations:** Department of Obstetrics and Gynecology, The Second Affiliated Hospital of Nantong University, Nantong, Jiangsu, China

**Keywords:** midwife-led continuity of care, individualized breast management, cesarean section, postpartum recovery, lactation function, complications, satisfaction

## Abstract

**Objective:**

The aim of this study was to investigate the effects of midwife-led continuity of care (MLCC) combined with individualized breast management on postoperative recovery, lactation function, and pelvic floor function in women undergoing cesarean section (CS).

**Methods:**

This quasi-experimental before-after study included 120 women who underwent CS between December 2022 and December 2024. Participants were randomly assigned to a control group or an intervention group, with 60 women in each group. The control group received routine perioperative care and the intervention group received a combined model of MLCC and individualized breast management. Additionally, breastfeeding education was emphasized, and breast management strategies were tailored to each participant’s breast condition. The primary outcome was exclusive breastfeeding at 48 h postpartum. Secondary outcomes included postoperative recovery indicators (time to ambulation, bowel movement, flatus, catheter removal, and wound healing), other measures of lactation function (time to lactation initiation, time to adequate lactation, and milk volume at 48 h), psychological status, pain level, sleep quality, breastfeeding self-efficacy, pelvic floor dysfunction, complications, care satisfaction, and quality of life.

**Results:**

The rate of exclusive breastfeeding at 48 h was higher in the intervention group than in the control group (56.67% vs. 38.33%). The intervention group showed significantly shorter times to first ambulation (*p* = 0.024), first bowel movement (*p* = 0.016), first defecation (*p* = 0.008), and urinary catheter removal (*p* = 0.014). Lactation function also improved significantly, with earlier initiation of lactation (*p* = 0.015), shorter time to adequate lactation (*p* < 0.001), and greater milk volume at 48 h postpartum (*p* < 0.001). In addition, the intervention group exhibited significantly lower scores on the Visual Analog Scale (VAS), Self-Rating Anxiety Scale (SAS), Self-Rating Depression Scale (SDS), and Pittsburgh Sleep Quality Index (PSQI) both during hospitalization and at the 3-month follow-up (all *p* < 0.05). Although the incidence of pelvic floor dysfunction showed a decreasing trend in the intervention group, this difference was not statistically significant (*p* > 0.05). However, the overall incidence of complications was significantly lower in the intervention group (10% vs. 25%, *p* = 0.031). Additionally, nursing care satisfaction score and quality of life were significantly improved in the intervention group.

**Conclusion:**

This study firstly reveals the impact of MLCC combined with individualized breast management for women delivering by SC. This care model may improve both postoperative recovery and lactation function, providing an emerging, effective care model for clinical maternity care with potential clinical applications.

## Introduction

Caesarean section (CS) is a common surgical procedure in the field of obstetrics, making it an effective tool for laboring women with obstructed labor and certain obstetric comorbidities, and plays an important role in ensuring the safety of parturient and infants (1). According to relevant reports, the rate of CS by CS in China has been as high as 36.7% (2), which is far beyond the 15% rate proposed by World Health Organization (WHO) (3). Across the globe, the rate at which CSs are performed for childbirth has gone up, with some countries seeing this rate reach 50% (4). While CS can be a life-saving procedure, it is associated with a range of postoperative challenges, including delayed recovery, acute and chronic pain, impaired lactation initiation, and higher risks of complications such as infection and pelvic floor dysfunction (5–7). These issues not only affect the physical health of mothers but also contribute to psychological distress, and reduced satisfaction (8).

In response to these challenges, various care models have been explored to improve postpartum outcomes. Among them, midwife-led continuity of care (MLCC) has emerged as an evidence-based model that provides coordinated, woman-centered care across the prenatal, intrapartum, and postnatal periods (9, 10). A quasi-experimental study conducted in Iran suggests that, in view of the positive impact of continuous team midwifery care on maternal and infant outcomes, this care model should be implemented in obstetric care systems-especially in countries with a high CS rate (10). A Cochrane’s systematic evaluation revealed that MLCC is an effective model of care that reduces adverse parturient and infant outcomes and promotes postoperative recovery, whether the labor is spontaneous or by CS (11). Ricchi et al. (12) showed that parturient-centered, MLCC leads to greater control of the parturient’s body throughout labor, which reduces anxiety and pain. The WHO also supports the widespread use of the MLCC model in perioperative and postoperative care for women in labor (13). Therefore, MLCC has been shown to be the best model of puerperal care for women at any level of risk (14). Similarly, breast management-including lactation support, breast massage, and personalized guidance-has been found to promote successful breastfeeding (BF), reduce breast-related complications, and improve milk output (15, 16). However, while both approaches have been studied independently, there is a notable lack of research investigating the combined effect of MLCC and individualized breast management on women undergoing CS. Therefore, the potential synergistic benefits of combining relational continuity (through MLCC) with specialized clinical support (through breast care) remain unexplored in the CS population.

Herein, this study aims to address this gap by evaluating the impact of a combined intervention-MLCC plus individualized breast management-on postoperative recovery, lactation function, pelvic floor health, and psychological well-being in women after CS. This study may provide women undergoing CS with a more clinically applicable model of care so as to reduce the discomfort of pregnancy.

## Methods

### Study design and setting

This quasi-experimental before-after study of 120 women undergoing CS in a randomized group with the aim of evaluating the improvement of postpartum recovery and lactation function in women undergoing CS by assisted MLCC combined with individualized breast management. This quasi-experimental study was conducted in accordance with the STROBE reporting guidelines, and was approved by the hospital ethics committee, and all study subjects and their families signed an informed consent form before enrollment.

### Participants

Women who underwent CS surgery in our hospital from December 2022–December 2024 were selected for the study. Inclusion criteria: (1) single fetus, full-term delivery; (2) elective CS surgery; (3) informed consent of the parturient and her family for this study. Exclusion criteria: (1) coagulation disorders; (2) malignant tumors; (3) cardiac, hepatic, renal, and other substantial organ dysfunction; (4) history of abdominal surgery; and (5) severe mental disorders.

### Allocation process

An independent researcher, not involved in patient recruitment, generated the random allocation sequence using a random number table. Participants were then assigned a unique serial number according to this sequence. To ensure concealment, the corresponding group assignments (Control or Intervention) for each serial number were placed in sequentially numbered, opaque, sealed envelopes (SNOSE). The enrolling midwife assigned each eligible consenting participant to a group by opening the next envelope in the sequence. Consequently, the first 60 participants assigned through this method formed the control group, and the subsequent 60 formed the intervention group.

### Blinding procedure

Due to the nature of the interventions, it was not possible to blind the participating women or the midwives providing care. However, to minimize assessment bias, the research staff responsible for collecting outcome data and the statistician performing the data analysis were blinded to group allocation. Throughout the study, these personnel were not involved in the delivery of care and had no access to information that could reveal a participant’s group assignment. Participants were advised not to discuss the details of their care with the outcome assessors.

### Sample size calculation

A sample size calculation was performed using PASS 11 software. With a significance level (*α*) of 0.05 (two-tailed), a power (1-*β*) of 0.80, and a medium effect size (d = 0.6), the analysis indicated a requirement of 52 patients per group. Accounting for an estimated 10% dropout rate, we aimed to recruit 60 patients per group, yielding a total sample size of 120.

### Intervention

The control group received routine perioperative care, which included standard preoperative education covering ward requirements, surgical steps, preoperative preparations, and perioperative precautions. Prior to surgery, vital signs, fetal heart rate, and uterine contractions were closely monitored, and a 6-h fasting period was observed. Intraoperative care involved adjusting operating room temperature and humidity as required. Postoperative management consisted of continuous vital sign monitoring, pain management (including instruction on analgesic pump and medication use), guidance on early feeding with specific timelines for resuming intake, and promotion of early mobilization such as turning in bed on the day of surgery, catheter removal at 24 h, and assisted ambulation. Incision care involved monitoring for bleeding or redness and keeping the wound clean and dry. Additionally, parturients were instructed in BF knowledge and techniques.

The intervention group received MLCC along with individualized breast management. A multidisciplinary team-composed of physicians, midwives (with team leaders having ≥5 years of experience), charge nurses, and dietitians-was established to deliver integrated care. Preoperative interventions included comprehensive health education using videos and manuals, psychological support to address fears related to pain and recovery, and the administration of 200–300 mL of glucose-sodium chloride solution 2–3 h before surgery. Perioperative measures focused on maintaining normothermia through prewarming the bed, using thermal blankets, and continuous temperature monitoring. Postoperative care involved a stepped pain management approach based on pain levels (incorporating education, non-pharmacological methods, patient-controlled analgesia, and oral analgesics), personalized dietary advancement with detailed guidance and record cards, early mobilization with massage and graded activity advice, support for BF including techniques for nipple and engorgement issues, and a structured pelvic floor exercise program followed by weekly follow-ups for 3 months after discharge. Supplementary Table 1 shows the comparison of nursing interventions between control and intervention groups.

### Outcomes

#### Primary outcome

Exclusive BF rate at 48 h postpartum.

#### Secondary outcomes

Postoperative recovery indicators (time to first ambulation, flatus, bowel movement, urinary catheter removal, wound healing), additional lactation outcomes (time to initiation, time to adequate lactation, and milk volume at 48 h), pain level, anxiety, depression, sleep quality, BF self-efficacy, feeding patterns, pelvic floor dysfunction, postoperative complications, care satisfaction, and quality of life.

Pain level was assessed at 1, 2, 3 d and 3 months postoperatively using a visual analog scale (VAS) with a total score of 10, with higher scores indicating greater parturient pain (17). The Self-Assessment Scale for Anxiety (SAS) and Self-Depression Scale (SDS) were completed before, after, and at 3 months, respectively. The SAS scale assessed the patients’ anxiety and the SDS assessed the patients’ depression. The higher the score, the more severe the degree of anxiety and depression. The Pittsburgh Sleep Quality Index (PSQI) (18) was used to assess the quality of parturient sleep, with a total of 18 scoring items, and the score was inversely proportional to the quality of sleep. The BF initiative included the Breastfeeding Self-Efficacy Scale score (BSES) (19) and the comparison of exclusive BF rate and mixed feeding rate, and the BSES score ranged from 14 to 70 points. The scale Cronbach’s *α* value was 0.927. The incidence of parturient incidence of pelvic floor dysfunction disorders such as pressure incontinence of urine (POP), and stress urinary incontinence (SUI) was recorded for the first 3 months postpartum. Parturient postoperative complications were recorded, including events such as gastrointestinal dysfunction, urinary retention, fever, incision infection, and breast tenderness. Patients’ satisfaction with nursing care was counted at discharge, and satisfaction with the nursing staff’s care was investigated using a homemade satisfaction questionnaire with a score of 100, with more than 85 indicating great satisfaction, 60–85 indicating basic satisfaction, and less than 60 indicating dissatisfaction. Comprehensive quality of life assessment questionnaire (GQOLI-74) (20) was used to comprehensively assess the quality of life of the two groups in the 3 months after surgery, the scale contains four dimensions such as somatic functioning, psychological functioning, social functioning, and material life status, the first three dimensions have scores ranging from 20 to 100, and the fourth dimension has scores ranging from 16 to 80, and the higher the scores indicate that the patients’ postoperative quality of life is better.

Postoperative complications within 3 months were recorded and included the following events:

*Fever*: Axillary temperature ≥ 38.5 °C on two consecutive measurements spaced 4 h apart, after the first 24 h post-surgery.
*Urinary retention*: Inability to void spontaneously after removal of the urinary catheter, requiring re-catheterization.
*Breast tenderness*: Patient-reported significant breast pain accompanied by hardness or swelling, interfering with breastfeeding or daily activity.
*Gastrointestinal dysfunction*: Presence of post-operative ileus, characterized by abdominal distension, nausea/vomiting, and absence of flatus or bowel movements beyond 72 h post-surgery.
*Incision infection*: Presence of purulent discharge from the surgical incision, with or without laboratory confirmation, requiring additional wound care or antibiotics.
*Intestinal adhesions*: Clinical diagnosis based on symptoms of chronic abdominal pain and bloating, with supporting imaging evidence if available.

Each event was captured on a dedicated case-report form and classified by the attending clinician; an independent obstetrician cross-validated every grade. Multiple distinct events per participant were counted separately (e.g., one woman could contribute both a Grade-1 fever and a Grade-2 wound infection); however, recurrent identical events (e.g., repeated temperature spikes during the same febrile episode) were considered a single adverse event of the highest grade achieved.

### Statistical analysis

In order to test whether there was any difference in the variables between the groups, quantitative analysis was performed using χ2 test in SPSS 21.0 according to the test conditions. All normally distributed continuous data were analyzed using Student’s *t*-test while non-parametric data were analyzed using Mann - Whitney U test. Count data and measured data are expressed as *n* (%) and mean ± SD, respectively, and all tests were two-sided. *p* < 0.05 was considered a statistically significant difference.

## Results

### Baseline characteristics of included participants

The 120 women who were included in the study had a mean age of 27.8 and a mean gestational week of 38.7, more than 70% had a medically indicated reason for CS, and more than 75% were pregnant for the first time. They were divided into Control and Intervention groups according to the requirements, and there were no statistical differences between the two groups in terms of preoperative baseline characteristics, intraoperative indications and neonatal characteristics, which were comparable (*p* > 0.05, Table 1).

**Table 1:** Baseline characteristics of participants.

Parameter		Control	Intervention	X^2^ (t)	*p*
Number		60	60	–	–
Age (years), mean ± SD		27.71 ± 4.98	28.04 ± 4.17	0.394	0.695
Week of pregnancy (weeks), mean ± SD		38.8 ± 1.42	38.64 ± 0.97	0.721	0.473
Prenatal BMI (kg/m^2^), mean ± SD		25.69 ± 2.38	25.88 ± 1.98	0.475	0.635
Indication for CS, *n* (%)	Medically indicated	47 (78.33)	44 (73.33)	0.409	0.522
	Maternity requirements	13 (21.67)	16 (26.67)		
Parturient history, *n* (%)	First pregnancy	50 (83.33)	47 (78.33)	0.484	0.487
	Multiple pregnancies	10 (16.67)	13 (21.67)		
Place of residence, *n* (%)	Town	42 (70.00)	47 (78.33)	1.087	0.297
	Village	18 (30.00)	13 (21.67)		
Educational level, *n* (%)	High school and below	13 (21.67)	11 (18.33)	0.797	0.671
	College or Bachelor’s Degree	35 (58.33)	33 (55.00)		
	Graduate students and above	12 (20.00)	16 (26.67)		
Duration of anesthesia (min), mean ± SD		62.24 ± 16.50	62.85 ± 17.20	0.198	0.843
Duration of surgery (min), mean ± SD		49.20 ± 16.30	49.05 ± 16.50	0.050	0.969
Estimated blood loss (mL), mean ± SD		292.00 ± 95.00	290.00 ± 92.00	0.117	0.907
Infant birth weight (g), mean ± SD		3279.00 ± 421.00	3326.00 ± 445.00	0.594	0.553
Sex of newborns, *n* (%)	Female	28 (46.67)	31 (51.67)	0.300	0.584
	Male	32 (53.33)	29 (48.33)		

Mean ± SD, mean ± standard deviation; BMI, body mass index; CS, caesarean section.

### Comparison of feeding patterns between groups

**Figure 1:**
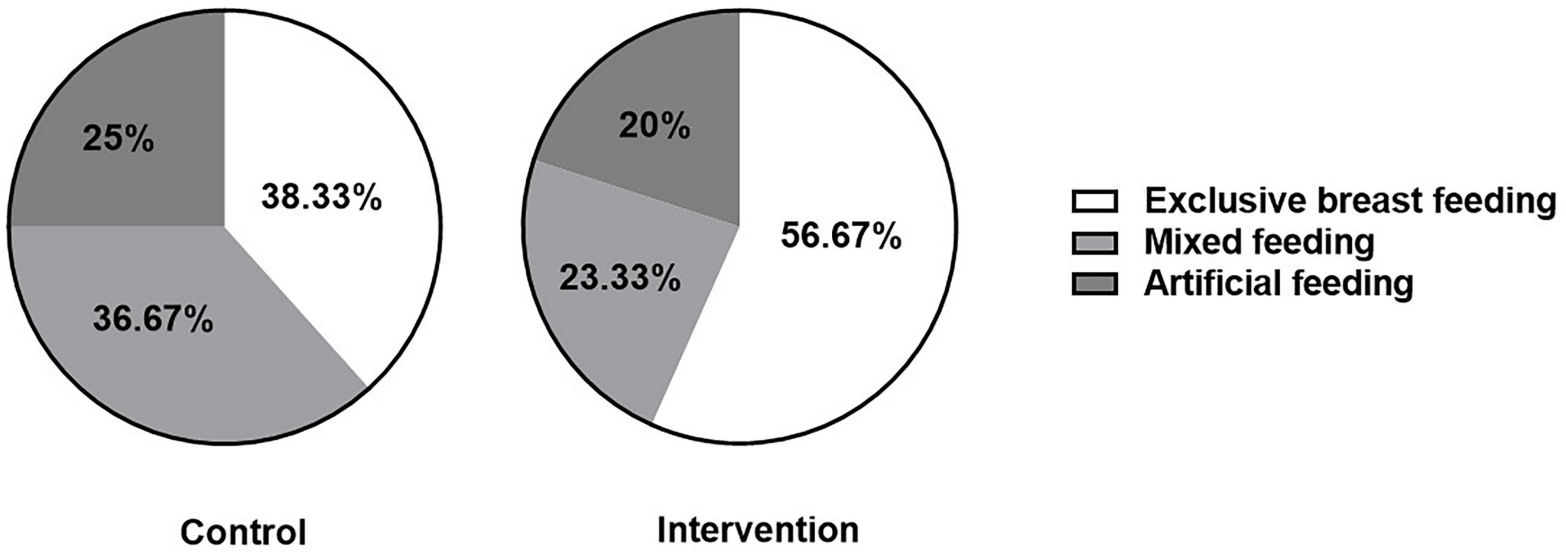
Comparison of feeding patterns between the two groups. BF, breastfeeding; sample size (n) = 60 per group.

In an exploratory analysis of feeding patterns, a higher proportion of parturients in the intervention group chose exclusive BF at 48 h postpartum compared to the control group (56.67% vs. 38.33%; absolute risk difference: 18.34%; 95% CI: 1.48 to 35.20; *p* = 0.044, Figure 1).

### Comparison of postpartum recovery outcomes between groups

From the results, postpartum recovery and lactation function were better in women given the intervention than in control women (Table 2). Among the indicators of postpartum recovery, the intervention group demonstrated significantly shorter times compared to the control group for the first time out of bed [33.95 ± 4.03 vs. 35.76 ± 4.6; mean difference (MD): −1.81; 95% confidence interval (CI): −3.36 to −0.26; *p* = 0.024], time to first bowel movement (44.57 ± 5.26 vs. 47.05 ± 5.86; MD: −2.48; 95% CI: −4.48 to −0.48; *p* = 0.016), time to first exhaust (23.62 ± 3.36 vs. 25.33 ± 3.55; MD: −1.71; 95% CI: −2.96 to −0.46; *p* = 0.008), and time to urinary catheter removal (8.25 ± 1.63 vs. 9.05 ± 1.87; MD: −0.80; 95% CI: −1.43 to −0.17; *p* = 0.014).

**Table 2:** Comparison of postpartum recovery and lactation outcomes between groups.

Outcome	Control	Intervention	X^2^ (t)	*p*
Number	60	60	–	–
Postpartum recovery				
The first time to out of bed (hours), mean ± SD	35.76 ± 4.60	33.95 ± 4.03	2.293	0.024
The first time to exhaust time (hours), mean ± SD	25.33 ± 3.55	23.62 ± 3.36	2.710	0.008
The first time to bowel movement (hours), mean ± SD	47.05 ± 5.86	44.57 ± 5.26	2.440	0.016
Urinary catheter removal time (hours), mean ± SD	9.05 ± 1.87	8.25 ± 1.63	2.498	0.014
Healing time of incision (days), mean ± SD	7.42 ± 1.95	6.77 ± 1.64	1.976	0.050
Lactation function				
Time to start lactation (hours), mean ± SD	30.96 ± 4.84	28.87 ± 4.47	2.457	0.015
Time to sufficient lactation (hours), mean ± SD	46.15 ± 2.88	42.62 ± 2.39	7.306	< 0.001
The amount of lactation at 48 h postpartum (mL), mean ± SD	139.00 ± 11.60	153.50 ± 12.25	6.657	< 0.001
48 h postpartum BF success rate, n (%)	36 (60.00)	45 (75.00)	3.077	0.079

Mean ± standard deviation. For recovery outcomes, a lower value (shorter time) indicates a better outcome. For lactation outcomes, a lower value indicates an earlier time to lactation, and a higher milk volume indicates a better outcome”.

### Comparison of lactation function outcomes between groups

In terms of lactation function, the time to start lactation (28.87 ± 4.47 vs. 30.96 ± 4.84; MD = −2.09; 95% CI: −3.75 to −0.43; *p* = 0.015) and the time to sufficient lactation (42.62 ± 2.39 vs. 46.15 ± 2.88; MD: −3.53; 95% CI: −4.46 to −2.60; *p* < 0.001) were significantly earlier in the intervention group. The amount of lactation at 48 h postpartum was also increased in the intervention group (153.5 ± 12.25 vs. 139 ± 11.6; MD = 14.50; 95% CI: 10.18 to 18.82; *p* < 0.001).

### Comparison of psychological and emotional scores (SAS, SDS, BSES) between groups

**Figure 2:**
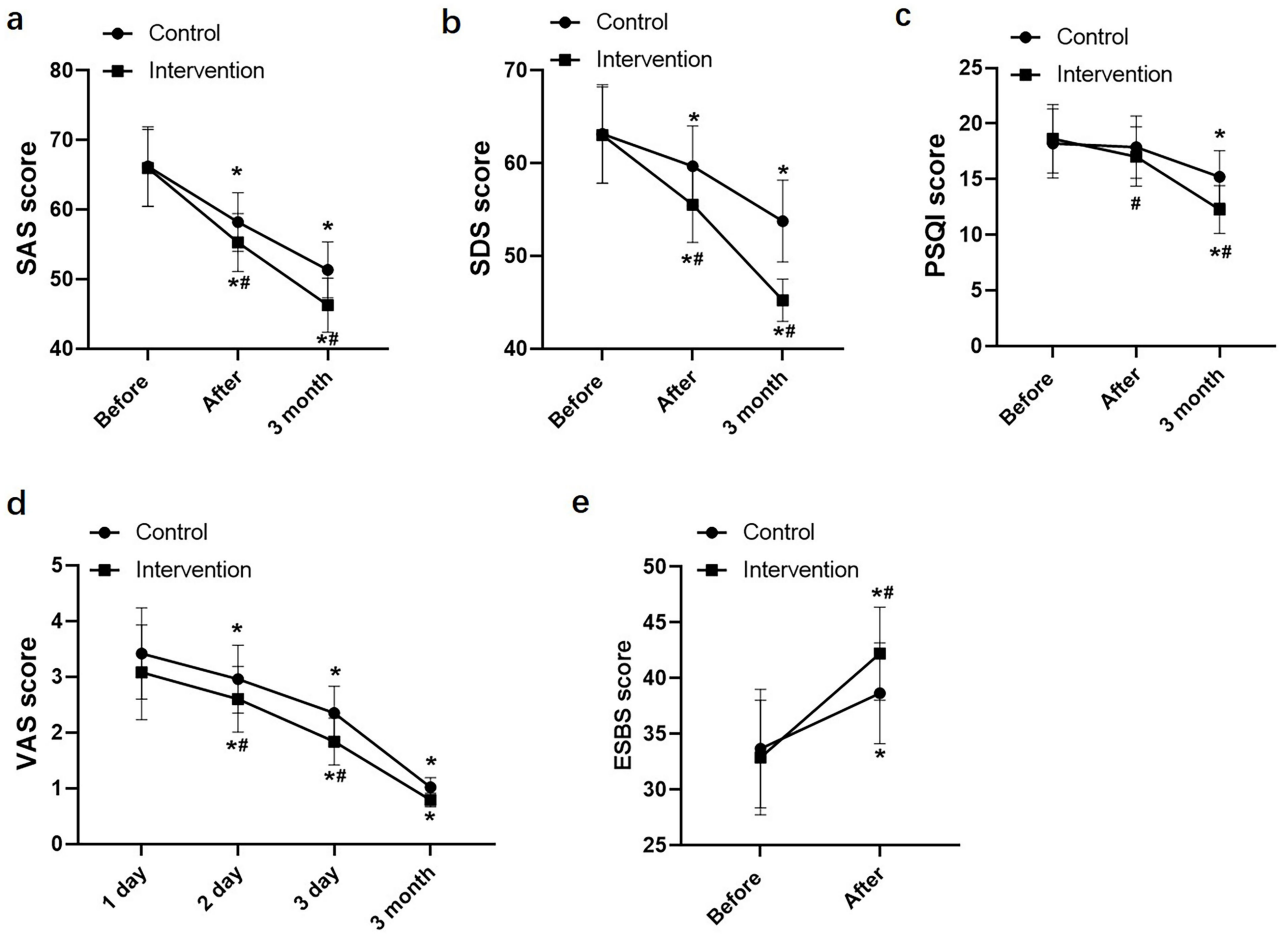
Comparison of maternal psychological and physical health indicators between the control and intervention groups before and after the intervention. **(a)** Self-Rating Anxiety Scale (SAS) scores; **(b)** Self-Rating Depression Scale (SDS) scores; **(c)** Pittsburgh Sleep Quality Index (PSQI) scores; (d) Visual Analog Scale (VAS) pain scores; **(e)** Breastfeeding Self-Efficacy Scale (BSES) scores. Data were presented as mean ± standard deviation. *p < 0.05, *compared before intervention; #*p* < 0.05, # compared with control; sample size *n* = 60 per group. Lower scores on VAS, SAS, SDS, and PSQI represent better outcomes. A higher score on BSES represents a better outcome.

Parturient SAS and SDS scores showed significant decreases, and the scores were lower in the intervention group, suggesting that anxiety and depression were relatively low (Figure 1). At the postoperative assessment, the mean SAS score in the intervention group was 2.94 points lower than in the control group (MD: −2.94; 95% CI: −4.50 to −1.38). This beneficial effect was even more pronounced at the 3-month follow-up (95% CI: −6.52 to −3.62). For SDS, a similar pattern was observed. Postoperatively, the intervention group’s SDS score was lower (MD: −4.15; 95% CI: −5.63 to −2.67). By the 3-month follow-up, this difference had widened substantially (MD: −8.54; 95% CI: −9.80 to −7.28). The ESBS scores showed a significant increase after the intervention, indicating that the intervention improved the confidence of the parturient to adhere to BF (Figure 2). The intervention group demonstrated a significant increase in confidence scores after the intervention compared to the control group (MD = 3.56; 95% CI: 2.09 to 5.03).

### Comparison of physical symptom scores (VAS, PSQI) between groups

In terms of postoperative pain assessment, VAS scores were lower in the intervention group, indicating better pain management (Figure 1). The MD of VAS scores between groups was −0.34 (95% CI: −0.64 to −0.04) on postoperative day 1, −0.36 (95% CI: −0.56 to −0.16) on day 2, and −0.51 (95% CI: −0.67 to −0.35) on day 3. The beneficial effect of the intervention on pain reduction persisted at the 3-month follow-up, with the Intervention group still reporting significantly lower pain scores (MD = −0.23; 95% CI: −0.27 to −0.19).

Sleep quality, as assessed by the PSQI, showed a marked improvement in the intervention group over time compared to the control group (Figure 2). While the difference between groups was not statistically significant immediately postoperatively (MD = −0.84; 95% CI: −1.79 to 0.11), a strong trend favoring the intervention was already evident. By the 3-month follow-up, the intervention group demonstrated a substantial and statistically significant improvement in sleep quality, with a mean PSQI score 2.93 points lower than the control group (MD: −2.93; 95% CI: −3.78 to −2.08).

### Exploratory analysis of pelvic floor dysfunction disorders and postoperative complications

Exploratory analysis of specific pelvic floor dysfunction disorders showed that the proportion of POP (18.83% vs. 8.33%; risk difference: −10.5%; 95% CI: −22.5 to 1.5%; *p* = 0.107) and SUI (11.67% vs. 3.33%; risk difference: −8.34%; 95% CI: −17.7 to 1.0%; *p* = 0.083) in the intervention group showed a decreasing trend compared to the control group, although these differences were not statistically significant (Table 3).

**Table 3:** Incidence of pelvic floor dysfunction disorders at 3-month follow-up in both groups.

Groups	POP	SUI
Control (*n* = 60), *n* (%)	11 (18.83)	7 (11.67)
Intervention (*n* = 60), *n* (%)	5 (8.33)	2 (3.33)
X^2^	2.596	3.003
*P*	0.107	0.083

POP, pressure incontinence of urine; SUI, stress urinary incontinence.

The incidence of postoperative complications such as urinary retention, gastrointestinal dysfunction and fever was lower (25% vs. 10%, risk difference: −15%; 95% CI: −27.8% to −2.2%; *p* = 0.031). An exploratory analysis of individual complication types is presented in Table 4.

**Table 4:** Postpartum complications within 3 months in both groups.

Groups	Urine retention	Fever	Breast tenderness	Gastrointestinal dysfunction	Incision infection	Intestinal adhesions	Total rates
Control (*n* = 60), *n* (%)	3 (5.00)	4 (6.67)	2 (3.33)	4 (6.67)	1 (1.67)	1 (1.67)	15 (25.00)
Intervention (*n* = 60), *n* (%)	1 (1.67)	2 (3.33)	1 (1.67)	1 (1.67)	1 (1.67)	0 (0.00)	6 (10.00)
X^2^							4.675
*p*							0.031

### Comparison of nursing satisfaction and quality of life scores between groups

**Figure 3:**
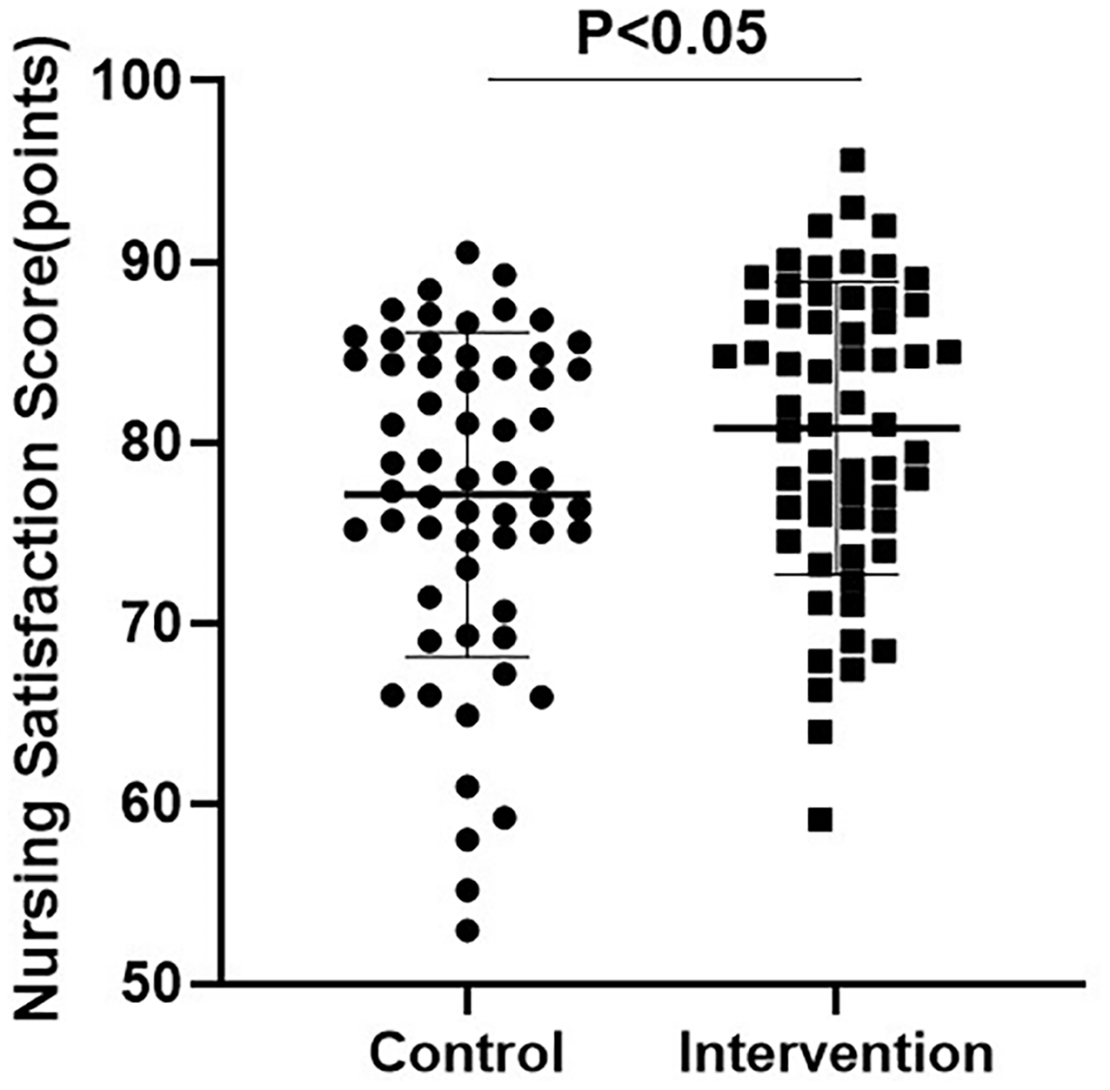
Maternal satisfaction scores with nursing care at discharge between two groups. Satisfaction was assessed using a 100-point questionnaire. Scores >85 indicate great satisfaction, 60–85 indicate basic satisfaction, and <60 indicate dissatisfaction; sample size (n) = 60 per group.

The results of the nursing satisfaction score indicated that the intervention group had a statistically significant higher nursing satisfaction score compared to the control group (MD: 3.67; 95% CI: 0.10 to 7.24; *p* = 0.020, Figure 3).

**Figure 4:**
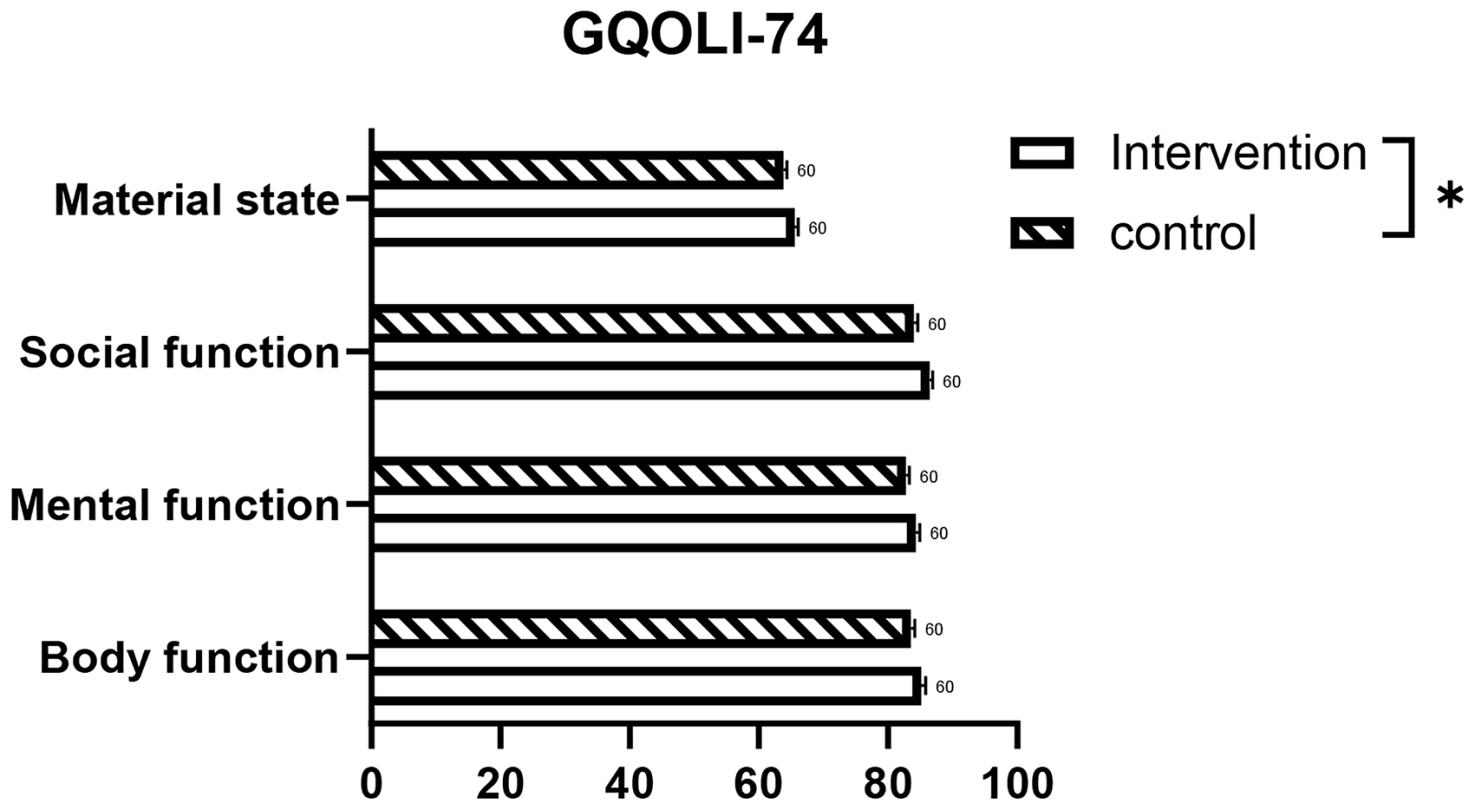
Scores of quality of life domains assessed by the GQOLI-74 questionnaire at the 3-month follow-up. Higher scores on all domains indicate a better quality of life. GQOLI-74, Generic Quality of Life Inventory-74. *p < 0.05; sample size (n) = 60 per group.

An exploratory analysis of the four GQOLI-74 subdomain scores revealed that women in the intervention group reported slightly higher values than those in the control group across body function, mental function, social function and material state (Figure 4). The effect sizes, expressed as MDs with their 95% CIs, were 1.59 (95% CI: 0.59 to 2.58; *p* = 0.002) for body function, 1.50 (95% CI: 0.54 to 2.46; *p* = 0.003) for mental function, 2.46 (95% CI: 1.60 to 3.32; *p* < 0.001) for social function, and 1.78 (95% CI: 0.88 to 2.68; *p* < 0.001) for material state (Supplementary Table 2).

## Discussion

### Summary

The WHO recommends MLCC in environments where the midwifery program is well-functioning, in order to ensure high-quality prenatal, perinatal and postnatal care (21). A recent study (22) predicts that in middle- and high-income countries, moderately expanding the intervention measures implemented by midwives (with a 10% increase in coverage every 5 years from 2020 to 2035) can prevent 26% of maternal deaths, 14% of fetal deaths, and 22% of neonatal deaths. Universal coverage (with a 95% increase in all intervention measures) can prevent 51% of maternal deaths, 47% of fetal deaths, and 44% of neonatal deaths. In addition to these life-saving estimates, the midwife-led care model has the greatest impact in disease prevention, by avoiding unnecessary medical interventions such as CSs, amniocentesis, episiotomy, instrumental delivery, more likely breastfeeding and natural vaginal delivery, as well as higher patient satisfaction (11, 23). A study was conducted on a larger scale, focusing on regional aspects and exploring the experiences and perspectives of midwives in low-income and middle-income countries regarding the midwife-led continuous care model (24). Another systematic review indicates that MLCC has a significant and positive impact on improving various maternal and infant outcomes in low-income and middle-income countries (25). The role of MLCC has been less explored in the Chinese population. More systematic and specific evidence is needed to understand the implementation of continuous care in various settings (26). In addition, there are fewer clinical data on the role of MLCC in postpartum recovery from CS. Herein, this study evaluated the effects of MLCC combined with individualized breast management on postoperative recovery, lactation function, and pelvic floor outcomes in women undergoing CS. The results showed that the primary outcome, exclusive breastfeeding at 48 h, was higher in the intervention group compared with the control group. The intervention significantly accelerated postoperative recovery, as indicated by shorter times to first ambulation, bowel movement, flatus, and urinary catheter removal. Lactation performance was markedly improved, with earlier initiation of lactation, reduced time to establish sufficient milk supply, and significantly increased milk volume at 48 h postpartum. Further results revealed substantial improvements in psychological well-being, including reduced anxiety and depression scores, enhanced BF self-efficacy, superior pain management, better sleep quality, higher nursing satisfaction and quality of life scores in the intervention group. Although the differences in specific pelvic floor dysfunction disorders did not reach statistical significance, a favorable trend was observed. The overall incidence of postoperative complications was significantly lower among women receiving the intervention. In conclusion, the combination of MLCC and individualized breast management represents an effective and promising care model for enhancing recovery and lactation outcomes in women after CS.

### Parturient postoperative recovery and lactation function

Earlier studies have focused on applying the MLCC model to investigate maternal and child health outcomes, notably the risk of CS. A U.S. labor and delivery data from 2009 to 2019 revealed that midwife-based delivery patterns decreased CS ratios (27). In Palestine, the implementation of the MLCC model has been associated with a lower proportion of unplanned CSs (28). In research that involved two cohort studies, utilizing data from the National Perinatal Audit Registry (PAN) and the Dutch National Perinatal Registry (PERINED), within the MLCC group, there were significantly lower rates of instrumental vaginal delivery and intrapartum CS (29). There are limited clinical data on the role of MLCC in postpartum recovery from CS. In this study, we compare the postoperative recovery and lactation function of women who received assisted MLCC combined with individualized breast management with those who received usual care. Parturient who received the specialized care model had earlier time to first bed, flatus, bowel movement and urinary catheter removal. A Danish study revealed that enhanced care for CS parturient reduced the length of hospitalization from an average hospital stay of 4 days to 2.4 days (30). This is in line with our findings that although the length of hospitalization was not counted in this study, there was a trend towards shorter time to removal of urinary catheter and time to first movement out of bed. Liu et al. (31) revealed that the length of hospitalization was also shorter for the first bowel movement of CS parturient who received psychosocial care and acupressure points. Overall, optimization of nursing management for CS parturient plays an important role in promoting parturient recovery. The service model advocated by midwives is maternity-centered and is a professional caregiver for low-risk pregnant women, who plays a key role in perinatal care, childbirth guidance, and gynecological disease prevention and health care, connecting the three stages of parturient prenatal, delivery, and postpartum periods, which can reduce medical interventions to a certain extent, and is important in promoting parturient postpartum recovery. There are fewer studies on the application of MLCC in CS, so we are more advocated to explore the effect of MLCC in CS to reduce postpartum stress in CS parturient.

Our results reveal that MLCC combined with individualized breast management improves lactation function in CS parturient, mainly in terms of increased lactation volume, earlier lactation time, and increased BF ratio. Lactation is a physiological process in which the mammary glands produce and secrete milk, and the lactation function of CS women may be affected by factors such as pain and physical discomfort, resulting in the inability to lactate normally and reduced willingness to breastfeed, which affects milk secretion and milk quality (32). In a study including 17 studies involving 18,533 randomized women, low-certainty evidence suggests that the MLCC model may have a positive impact on BF initiation, though this effect did not reach statistical significance (33). A study that recruited 1730 pregnant women in 9 hospitals in Shanghai found that MLCC increased the rate of BF within the first 24 h (34). A Korean study developed a targeted care program for BF and found that CS women who received breast massage and BF education had higher rates of BF and less breast swelling (35). A clinical study in China assigned individualized interventions for CS parturient, and parturient BF rates, feeding satisfaction and duration were higher (16). Therefore, effective nursing interventions are necessary to increase the willingness and rate of parturient BF in CS. In addition, Eker et al. (36) found that a rational model of lactation management improves BF success and reduces the incidence of breast problems. Therefore, the combination of the two interventions is beneficial for the improvement of parturient lactation function in CS.

### Mechanisms underlying improved outcomes: bridging psychosocial support and physiology

The superior outcomes observed, particularly in lactation, likely stem from a synergistic interplay between psychological and physiological mechanisms. The MLCC model provides continuous, empathetic psychosocial support, which is known to mitigate stress, anxiety, and pain (37). This reduction in maternal stress is crucial, as psychological distress can elevate cortisol levels, which antagonizes the milk-ejection reflex and may suppress prolactin secretion (38). By offering reassurance, education, and hands-on assistance, our intervention potentially reduced stress-related physiological inhibition. Furthermore, the individualized breast management component, including techniques like massage and proper latch guidance, provides direct physical stimulation. This stimulation is a key trigger for prolactin release and oxytocin-mediated milk ejection, thereby enhancing milk synthesis and flow (39). Thus, the combination of stress reduction (favoring hormonal milieu) and physical stimulation (directly activating lactation) offers a plausible biopsychosocial mechanism for our findings.

### Psychological and emotional scores (SAS, SDS, BSES)

Our findings indicated that parturient who received specialized care had significant reductions in depression, and anxiety. The results of a qualitative assessment of pregnant women in the UK revealed that the MLCC model enhanced parturient self-confidence and demonstrated high levels of satisfaction (40). Several studies have developed different models of care for women with CS, and data have shown that they are able to reduce negative emotions and complications in women with CS compared to usual care (41). A randomized controlled trial (RCT) conducted between May 2012 and June 2013, which enrolled women in their second trimester of pregnancy, showed that the incidence of postpartum “birth - related flashback symptoms” was significantly lower in the intervention group (midwife psycho-education intervention) than in the control group (42). This finding indicates that the intervention effectively alleviated negative emotions associated with birth trauma.

Moreover, negative emotions have an important regulatory role in promoting lactation function and postpartum recovery, and as shown in the previous results, postpartum recovery and lactation function were improved in CS parturient under this intervention model, in large part due to the reduction of anxiety and depression.

### Physical symptom scores (VAS, PSQI)

Postoperative pain in CS is mainly due to direct incision damage to nerve endings as well as inflammatory response caused by tissue damage, and also includes various sources of pain such as postpartum uterine contraction pain, BF pain, back pain, and so on (43). Postoperative pain can adversely affect both the CS parturient herself and the newborn. In this study, the VAS scores of women who received specialized care were lower than the control. Hunter et al. (44) revealed that music intervention can reduce anxiety and pain associated with CS in the short term. In a study encompassing 15 studies and comparing the effectiveness of MLCC with other care models for mothers and their infants, women receiving midwife-led continuity of care were less likely to undergo regional analgesia (11). The significant improvement in PSQI scores at the 3-month follow-up indicates better sleep quality, which is often severely disrupted postpartum. This improvement is likely a secondary benefit of better pain management, reduced infant feeding difficulties due to successful lactation, and lower overall anxiety levels.

### Incidence of pelvic floor dysfunction disorders and complications

Here, the incidence of pelvic floor dysfunction disorders, including POP and SUI, was reduced in women who received transfer care, and there was a significant reduction in postoperative complications. The incidence of pelvic floor disorders was reduced in SC parturient compared to vaginal delivery, but the physiologic act of pregnancy still increases the probability of pelvic floor disorders (45). Pelvic floor muscle training during and after pregnancy has a gainful effect on improving pelvic floor muscle strength and reducing pelvic floor dysfunction disorders (46). In addition, postoperative complications of CS including wound infection and urinary retention can negatively affect long-term parturient health. Effective nursing practices are effective in avoiding postoperative complications, which has been demonstrated in several studies (47). In addition, parturient satisfaction with the care model of MLCC combined with individualized breast management was better in comparison, and parturient quality of life scores were higher after 3 months postoperatively, which is consistent with the results of other nursing interventions (48, 49).

### Clinical implications and challenges

The implementation of our combined model holds significant promise for improving maternity care globally. A key advantage of the MLCC component is its potential for cost-effectiveness and scalability, even in resource-constrained settings. Training and empowering midwives to lead coordinated care requires investment in human resources rather than expensive technology. This model can potentially reduce downstream costs by decreasing complication rates, shortening hospital stays, and reducing the need for management of lactation failures. Successful implementation would involve integrating principles of continuity into existing healthcare frameworks, task-sharing, and providing specialized training for midwives in breast management techniques. The use of structured protocols and group education sessions, as done in our study, can optimize resource use. Future implementation research should focus on adapting and testing this model in diverse socioeconomic contexts to develop tailored strategies for scaling.

However, legitimate concerns have been raised regarding the potential negative impact of midwife-led continuity models on midwives themselves, including increased out-of-hours availability, inadequate staffing levels, and difficulty maintaining work-life balance (50, 51). Additionally, cultural and socio-economic factors significantly influence the acceptability and successful adoption of midwife-led continuity of care (52). In settings where traditional medical authority is highly valued, some women and families may initially perceive midwife-led models as subordinate to obstetrician-led care, potentially affecting engagement. Economic constraints, limited health literacy, and lack of family support can further hinder participation, particularly in low-resource communities.

### Strengths and limitations of the study

As a pioneering mixed-methods study, this study provides comprehensive insights into postpartum recovery and lactation function. However, several limitations must be considered. First, this study was a quasi-experimental before-after design rather than a randomized controlled trial, the possibility of selection bias cannot be completely excluded. Second, this study was conducted at a single center with a relatively small sample size, which may limit the generalizability of our findings to broader populations and other healthcare settings. While adequate for our pre-specified primary analysis, small sample size precluded us from performing reliable multivariable regression analyses to adjust for potential baseline confounder. Therefore, the results of this study should be interpreted as demonstrating an unadjusted effect, and future larger trials with sufficient power for adjusted analyses are warranted to confirm our findings. The results may also be influenced by the specific cultural context of perinatal care in China, which could affect the transferability of the intervention model to other cultural settings. Third, while we assessed a wide array of endpoints to capture the intervention’s multidimensional impact, the absence of formal multiple-comparison adjustments increases the possibility that some statistically significant findings represent type I errors. Consequently, the reported *p*-values, especially for secondary outcomes, should be interpreted as indicative and require confirmation in future dedicated trials. Fourth, despite collecting data on key lactation metrics, our follow-up period did not extend beyond 3 months for most outcomes. This precludes any analysis of the intervention’s effect on long-term BF duration and sustainability, which are critical indicators of lactation success. Fifth, although outcome assessors were blinded, the nature of the psychosocial intervention made full blinding of participants and care providers impossible. This introduces a potential for performance and reporting bias, particularly for patient-reported outcomes (e.g., satisfaction, pain scores), where expectations may influence responses. Sixth, another limitation is the use of a non-validated, homemade questionnaire to assess nursing care satisfaction. While this tool was designed to be highly relevant to the specific interventions studied, the lack of formal validation for its psychometric properties (e.g., construct validity, internal consistency) should be considered when interpreting the satisfaction results. Finally, as the sample size calculation was not based on the now-defined primary outcome, the findings should be interpreted as exploratory. Despite these limitations, we believe our exploratory findings offer robust and clinically relevant signals. This study serves as a foundation for future large-scale, multi-center, longer-term randomized controlled trials with pre-specified statistical hierarchies to conclusively validate the efficacy of this integrated care model.

## Conclusion

Our findings suggest that the care model of MLCC combined with individualized breast management may promote postoperative recovery and lactation function in women undergoing CS. This model shows promise as a potentially effective clinical care model, but its efficacy needs to be confirmed in larger, randomized controlled trials.

## Data Availability

The datasets presented in this study can be found in online repositories. The names of the repository/repositories and accession number(s) can be found in the article/Supplementary material.
