# Supplementary figures and images for: Effect of midwife-led continuity of care combined with individualized breast management on postpartum recovery and lactation function in women undergoing cesarean section
Source: Front Med (Lausanne). 2025 Oct 31;12:1608027. doi: 10.3389/fmed.2025.1608027 (PMC12615375; doi:10.3389/fmed.2025.1608027)

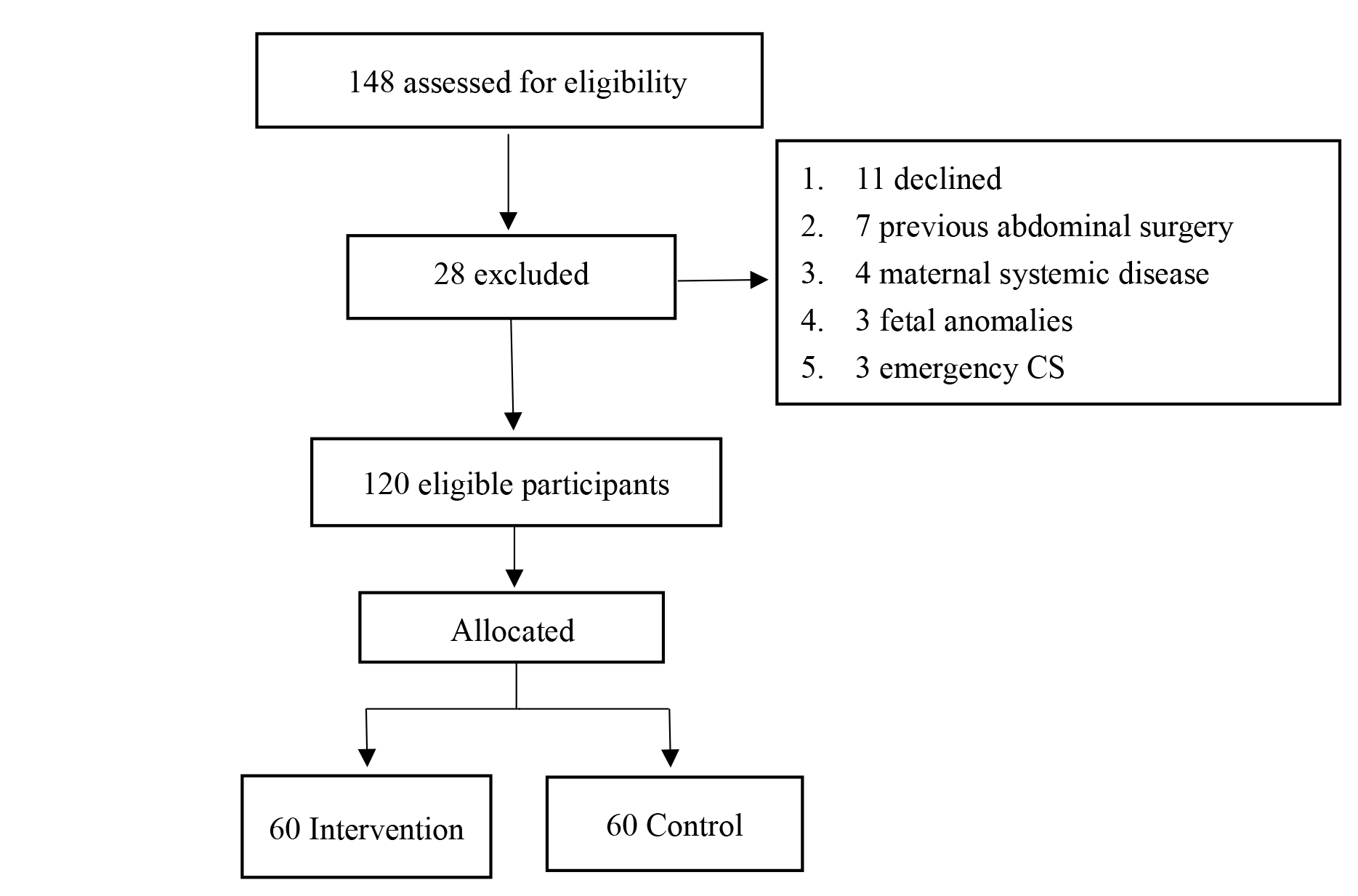

Supplement: Supplementary file 1 [file Image_1.TIF]
