# Supplementary material for: Effect of midwife-led continuity of care combined with individualized breast management on postpartum recovery and lactation function in women undergoing cesarean section
Source: Front Med (Lausanne). 2025 Oct 31;12:1608027. doi: 10.3389/fmed.2025.1608027 (PMC12615375; doi:10.3389/fmed.2025.1608027)
Supplement: Supplementary file 2 [file Table_1.DOCX]

**Supplementary Table 1 Comparison of nursing interventions between control and intervention groups**

| **Nursing domain** | **Control group (routine care)** | **Intervention group (MLCC + Individualized breast management)** |
| --- | --- | --- |
| **Team structure** | Standard ward nursing staff | A dedicated multidisciplinary team (physicians, experienced midwives [≥5 years], charge nurses, dietitians). Midwife leader coordinates care and ensures quality |
| **Preoperative care** | Basic health education: ward rules, surgical procedure, preoperative preparations, perioperative precautions; arturient vital signs, fetal heart rate and contractions were closely observed before the operation, and fasting and water restriction were imposed for 6 h | **Proactive and comprehensive education:** Team introduction & structured education (videos, manuals) **Psychological intervention:** Addressing fears of pain, explaining recovery process, correcting misconceptions; **Preoperative hydration:** 200-300 mL glucose-sodium chloride solution 2-3 h before surgery; |
| **Intraoperative care** | Adjust operating room temperature/humidity as required. | **Active warming protocol:** Pre-warmed bed and bedding Use of thermal blankets; Pre-warming of contact instruments;  Continuous maternal temperature monitoring |
| **Postoperative: pain management** | Instruction on analgesic pump and medication use. | **Stepped, multimodal pain management:** **Mild pain:** Non-pharmacological methods (education, distraction, music therapy, infrared therapy); **Moderate pain:** Increased use of patient-controlled analgesia; **Severe pain:** Oral analgesics added; **Pain reassessment and plan adjustment every 4h or as needed** |
| **Postoperative: diet management** | Guidance on timing for resuming water and food intake. | **Personalized and structured dietary plan:** Video and verbal guidance; **Use of a dietary record card;** Clear protocol: sips of water within 12h → liquids → regular diet after first defecation, avoiding gas-producing foods |
| **Postoperative: activity/mobility** | Encourage turning in bed, remove catheter at 24h, advise on ambulation. | **Proactive and assisted mobilization:** **Early (0-6h):** Massage of back and limbs by nurses/family to promote circulation; **Graded exercise:** Personalized guidance on positioning, leg exercises, catheter removal, and ambulation based on pain and tolerance; |
| **Breast management & bf support** | Instruct on basic breastfeeding knowledge and skills | **Enhanced and individualized support:** **Education:** Advantages and techniques of breastfeeding, early skin-to-skin contact; **Assessment & Intervention:** For inverted nipples (nipple pulling exercises), for engorgement (professional breast massage); Active encouragement to practice breastfeeding |
| **Follow-up & Rehabilitation** | / | **Structured postnatal functional exercise program:** **Personalized pelvic floor muscle training plan;** **Regular follow-up:** Weekly telephone/outpatient follow-ups for 3 months to guide and adjust exercise intensity. |

Notes: MLCC: midwife-led continuity of care.
