# Supplementary material for: Effect of midwife-led continuity of care combined with individualized breast management on postpartum recovery and lactation function in women undergoing cesarean section
Source: Front Med (Lausanne). 2025 Oct 31;12:1608027. doi: 10.3389/fmed.2025.1608027 (PMC12615375; doi:10.3389/fmed.2025.1608027)
Supplement: Supplementary file 3 [file Table_2.DOCX]

**Supplementary Table 2 Mean GQOLI-74 scores at 3-month follow-up**

| **GQOLI-74 Subdomain** | **Control Group (n=60),** Mean ± SD | **Intervention Group (n=60),** Mean ± SD | **Mean Difference (Intervention-Control)** | **95% CI for Difference** | **P** |
| --- | --- | --- | --- | --- | --- |
| Body Function | 83.54 ± 2.16 | 85.13 ± 2.65 | 1.59 | 0.59 to 2.58 | 0.002 |
| Mental Function | 82.76 ± 2.18 | 84.26 ± 2.55 | 1.50 | 0.54 to 2.46 | 0.003 |
| Social Function | 83.96 ± 2.38 | 86.42 ± 1.92 | 2.46 | 1.60 to 3.32 | <0.001 |
| Material Life State | 63.78 ± 2.04 | 65.56 ± 2.18 | 1.78 | 0.88 to 2.68 | <0.001 |

Note: SD = Standard Deviation; CI = Confidence Interval. Higher scores indicate better quality of life.
